# Bioinspired Co-Assembled Hydrogels Constructed from Marine Self-Assembling Peptides and Polyphenol Network: Antioxidant and Infected Wound Healing

**DOI:** 10.3390/antiox14070785

**Published:** 2025-06-26

**Authors:** Chuhan Wang, Dingyi Yu, Wen Liu, Xiang Zhu, Hanzhe Zhang, Shuang Zheng, Jingdi Chen

**Affiliations:** Marine College, Shandong University, Weihai 264209, China; 202200700202@mail.sdu.edu.cn (C.W.); 202000810156@mail.sdu.edu.cn (D.Y.); 202300700197@mail.sdu.edu.cn (W.L.); 202300810207@mail.sdu.edu.cn (X.Z.); 202300700270@mail.sdu.edu.cn (H.Z.); 202100810142@mail.sdu.edu.cn (S.Z.)

**Keywords:** bioinspired hydrogel, self-assembling peptides, antioxidant activity, infected wound healing

## Abstract

Infectious wounds pose formidable clinical challenges due to hypoxia, exacerbated inflammation, and persistent microbial colonization. To address this, we developed a bioinspired multifunctional hydrogel (PTDPs) through the in situ freeze-thaw co-assembly of polyvinyl alcohol (PVA), tea polyphenols (TP), polydopamine (PDA), and marine-derived self-assembling peptides (AAPs). The resultant PTDP hydrogel formed an intricate hydrogen-bonded network that enhanced mechanical robustness and substrate adhesion. TP and PDA synergistically confer potent antioxidant properties: TP scavenges radicals via phenolic hydroxyl groups while PDA enhances responsiveness to diverse radicals in hypoxic environments. Integrated with AAPs’ pro-regenerative functions and PDA’s broad-spectrum antimicrobial efficacy, this system generates therapeutic synergy. Characterization revealed outstanding physicochemical properties including tunable plasticity, high swelling ratios, and sustained hydration retention. In vitro studies demonstrated potent antioxidant activity, efficient inhibition of *Staphylococcus aureus* and *Escherichia coli* proliferation, and cytocompatibility facilitating endothelial cell migration/proliferation. In murine full-thickness infected wound models, the PTDP hydrogel significantly accelerated wound closure, enhanced neovascularization, and improved collagen deposition, underscoring its potential as an innovative therapeutic platform for infected and chronic wounds with strong translational prospects.

## 1. Introduction

Wound healing is a multistage biological process, yet infectious wounds are a major clinical challenge [1,2,3]. Hypoxia restricts oxygen supply which is vital for healing, pathogen-induced inflammation disrupts the healing cascade, and microbial colonization damages tissues, leading to delayed healing and potential complications [4,5,6]. Moreover, oxidative stress, often overlooked, is a significant hurdle. Wounding triggers an imbalance between reactive oxygen species (ROS) production and antioxidant defenses, exacerbated in infectious wounds by microbial metabolism and immune activation. Elevated ROS damage cellular components, impede cell functions, and hinder angiogenesis by inactivating growth factors [7,8].

Bioinspired materials have emerged as a promising solution in the field of tissue engineering and regenerative medicine. By mimicking the natural extracellular matrix (ECM) and biological processes, these materials can provide a more favorable microenvironment for cell growth and tissue repair. Recent research has focused on creating bioinspired materials that can not only physically support tissue regeneration but also actively interact with cells through bioactive cues [9,10]. For instance, biomimetic scaffolds designed to replicate the hierarchical structure and biochemical properties of the ECM have shown enhanced cell adhesion and differentiation capabilities [11]. Among various biomaterials, hydrogels, with their high-water content, tunable mechanical properties, and ability to encapsulate bioactive molecules, have shown great potential in wound-healing applications. Hydrogels can closely mimic the physiological environment of tissues, facilitating cell–matrix interactions and nutrient diffusion. A recent meta-analysis of 50 clinical trials demonstrated that hydrogel-based dressings significantly reduced wound healing time by an average of 3.2 days compared to traditional dressings [12].

Polydopamine (PDA), inspired by the adhesive properties of mussel foot proteins, has attracted extensive attention due to its strong bio-adhesiveness and broad-spectrum antimicrobial activity. PDA can adhere to various surfaces, including both hydrophilic and hydrophobic materials, through a combination of covalent and non-covalent interactions [13]. In addition to its adhesive properties, PDA has been shown to generate reactive oxygen species (ROS) under certain conditions, which can inhibit the growth of bacteria [14]. Marine-derived self-assembling peptides (AAPs), on the other hand, can spontaneously form nanostructured scaffolds similar to the ECM, promoting cell adhesion, proliferation, and tissue regeneration. AAPs often contain specific amino acid sequences that can interact with cell surface receptors, triggering intracellular signaling pathways related to tissue repair [15]. Polyvinyl alcohol (PVA) and tea polyphenols (TP) are also well-known for their excellent biocompatibility and antioxidant properties, respectively. PVA has been widely used in the preparation of hydrogels due to its ease of processing and good mechanical properties [16]. TP, rich in phenolic hydroxyl groups, can scavenge free radicals and reduce oxidative stress at the wound site, thereby protecting cells from damage and promoting tissue repair [17].

Despite the individual advantages of these components, integrating them to create a multifunctional hydrogel with balanced mechanical, adhesive, and biological properties remain a challenge. Previous attempts at combining these materials have often resulted in compromised performance, such as reduced mechanical strength or insufficient biological activity. In this study, we report the development of a bioinspired co-assembled hydrogel (PTDP) combining PVA, TP, PDA, and AAPs through an in-situ freeze-thaw process. The aim is to create a material that can simultaneously address the multiple challenges in infectious wound healing, such as providing mechanical support, promoting tissue regeneration, and combating microbial infections. The in-situ freeze-thaw process enabled the formation of a unique network structure that optimized the synergistic effects of each component, including enhancing the antioxidant capabilities of the hydrogel. This study not only contributes to the development of advanced wound healing biomaterials but also explores the potential of supramolecular assembly strategies in biomedical applications, especially in relation to antioxidant functions, providing new insights for the design of multifunctional biomaterials.

## 2. Materials and Methods

### 2.1. Materials

AAPs (prepared using a method described previously by our research group [15]), polyvinyl alcohol-type 1799-(PVA) (degree of alcoholysis: 98–99% (mol/mol)) was purchased from Shanghai Aladdin Biochemical Technology Co., Ltd. (Shanghai, China). Dopamine hydrochloride (DA, 98%) was purchased from McLean Biochemical Technology Co., Ltd. (Shanghai, China). *Staphylococcus aureus* (*S. aureus*, ATCC 6538) and *Escherichia coli* (*E. coli*, ATCC 25922) were purchased from the China Conservation Center (Wuhan, China). HUVEC cells were obtained from the Kunming Cell Bank, Chinese Academy of Sciences (Kunming, China). A CCK-8 kit was purchased from APEX BIO, Inc. (Cambridge, MA, USA). An AO/EB staining kit was acquired from Shanghai Sangong Biotechnology Co., Ltd. (Shanghai, China). The materials used throughout this study were of analytical pure grade and applied directly.

### 2.2. Preparation of AAPs

AAPs were prepared based on our previously reported method [15]. The *Azumapecten farreri* meats were washed, drained, and ground before being dissolved in water at a ratio of 1:3 (mantle:water). The hydrolysis reaction was carried out using animal proteases, i.e., 1000 U/g (raw material), at 53 °C and pH 7 for 3–5 h while stirring was carried out. Next, the mixture was incubated in boiling water for 10 min and cooled immediately to inactivate the enzymes. Following inactivation, the hydrolysate was fractioned into <3 K Da group using an ultrafiltration system (GCM-S-02L, GUOCHU Technology, Xiamen, China). The ultrafiltration membranes (GC-UF0031made of PES, GE Healthcare, Chicago, IL, USA) and AAPs were obtained by rotary evaporation (N-1300V, EYELA, Tokyo, Japan) and freeze-drying (FDU-2110, EYELA, Tokyo, Japan) in sequence.

### 2.3. Preparation and Characterization of Hydrogels

A certain amount of DA was weighed and dissolved in 10 wt% PVA solution, and using a 1 mol/L NaOH solution, the pH of the PVA solution was set to 10.0. A water bath at 50 °C for 12 h was used to obtain a PDA/PVA black solution. A specified amount of AAPs was weighed and added to 10 wt% PVA solution, stirred thoroughly, and ultrasonicated (40 kHz) at 40 °C for 30 min to remove air bubbles to obtain an orange transparent solution of AAPs/PVA.

The solution was dispensed into custom-designed acrylic molds and subjected to freezing at −80 °C for 4 h, before being thawed at room temperature for 2 h. This process was repeated three times to yield a hydrogel. Among them, the PVA solution was obtained as PVA hydrogel by the low-temperature freeze-thaw method [18]. The PVA solution and the TP solution were mixed homogeneously in a certain ratio to obtain the TP precursor liquid. The TP precursor liquid was obtained as the PT hydrogel by the low-temperature freeze-thawing method. The precursor solution was immersed in the prepared PDA/PVA solution at room temperature for 12 h. PTD hydrogel was synthesized via the cryo-freeze-thaw method. The AAP/PVA solution was mixed with the TP solution in a certain ratio and soaked in the prepared PDA/PVA solution at room temperature for 12 h. The PTDP hydrogel was obtained by the low-temperature freeze-thaw method.

The ratio of PVA to TP is key to determining whether the hydrogel can be formed into a gel. The ratio of PVA to TP was set to 1:0.5, 1:0.75, 1:1, and 1:2, respectively, the time of gel formation was recorded, and photographs were taken to observe the formation of the gel.

### 2.4. Characterization of Hydrogels

#### 2.4.1. Fourier Transform Infrared Spectroscopy (FT-IR)

FT-IR (IRSpirit-T, Shimadzu, Kyoto, Japan) was used for the determination, with a resolution of 400–4000 cm^−1^ [19,20].

#### 2.4.2. Scanning Electron Microscope (SEM)

The fully dehydrated hydrogel particles were ground into powder, mounted onto double-sided conductive tape, and sputter-coated with gold under vacuum for SEM observation. The samples were gold-sprayed under vacuum, with an accelerating voltage set to 12.5 kV [19,20].

#### 2.4.3. Plasticity and Adhesion

Hydrogel precursors were extruded into specific molds of different shapes and then frozen and thawed at low temperatures. Photographs were taken to observe the plasticity of the hydrogels. Effective wound dressings should adhere firmly to cutaneous tissues while providing a protective barrier against external irritants. Hydrogels are tested for adhesion to materials with different substrates, such as plastic, steel, and skin [21].

#### 2.4.4. Swelling Ratio

The initial weight of the hydrogel was calculated and recorded as *W_d_*. The samples were soaked in phosphate-buffered saline (PBS) at different pH values. The weights of the hydrogels in the solution were measured as *W_s_* at the presupposed time point [21]. The swelling rate was calculated using Equation (1):

(1) $$Swelling\;rate\;\left(\%\right)=\frac{W_{s}}{W_{d}}\times 100$$

where *W_d_* represents the initial dry weight of the hydrogel, and *W_s_* corresponds to its swollen weight.

#### 2.4.5. Water Retention Rate

The same mass of hydrogel was placed in PBS (pH = 7.4), and a specific period was selected for weighing and calculating the water retention according to Equation (2) [22]:

(2) $$Water\;retention\;rate\;\left(\%\right)=\frac{W_{S}-W_{d}}{W_{i}-W_{d}}\times 100$$

where *W_s_* represents the swollen hydrogel mass at a specific time, *W_d_* denotes the weight of the hydrogel after freeze-drying, and *W_i_* corresponds to the equilibrium swollen mass.

#### 2.4.6. Mechanical Properties

The hydrogels were sectioned into rectangular prisms (20 mm length × 35 mm width × 6 mm thickness) and placed in the universal material testing machine to test their tensile properties, with the tensile speed set at 90 mm/min. Additionally, the hydrogel samples were cut into cylinders of 35 mm diameter and 6 mm thickness, placed in the universal material testing machine, and compressed twice by using a 75 mm flat plate, with a deformation variable of 70% and a compression speed of 60 mm/min [23].

### 2.5. In Vitro Antioxidant Activity

#### 2.5.1. ABTS Free Radical Scavenging Capacity

ABTS^+^ solution was prepared by mixing 0.7 mM ABTS with an equimolar volume of 2.45 mM potassium persulfate solution. This ABTS^+^ solution was then incubated at room temperature, shielded from light, for a duration of 16 h. Following incubation, the solution was diluted with phosphate buffer (5.0 mM, pH 7.4) to achieve an optical density (OD) value of 0.70 ± 0.05 at a wavelength of 734 nm. Following dilution, 5.0 μL of the ABTS^+^ solution was added to 200 μL of each hydrogel group extract and incubated for 10 min at room temperature (25 ± 1 °C, in a dark environment), and the OD values of the mixtures were measured by an enzyme meter at 734 nm. Phosphate-buffered saline (PBS) served as the blank control [24].

#### 2.5.2. DPPH Free Radical Scavenging Capacity

For DPPH radical scavenging assessment, 2 mL aliquots of each hydrogel extract were combined with 2 mL of 0.1 mM DPPH solution (95% methanol), protected from light, and incubated at ambient temperature (25 ± 1 °C) for 30 min. Absorbance at 517 nm was subsequently quantified using an enzyme meter [24].

#### 2.5.3. PTIO Free Radical Scavenging Capacity

The freeze-dried hydrogels were immersed in 2 mL of PTIO solution and incubated at 37 °C for 90 min under light-protected conditions. The supernatant’s absorbance was subsequently measured at 557 nm using an enzyme meter. The same mass of PBS was used instead of the experimental group hydrogel as a blank matrix [24].

### 2.6. In Vitro Antimicrobial Activity

#### 2.6.1. Inhibition Rate

A 150 μL aliquot of bacterial suspension (initial concentration: 1 × 10^8^ colony-forming units (CFU)/mL, determined by CFU plating) was inoculated into 200 mL of sterile Luria-Bertani (LB) liquid medium (composed of 10 g/L tryptone, 5 g/L yeast extract, and 10 g/L NaCl), then cultured aerobically at 37 °C with shaking (180 rpm) for 24 h. The 24-h culture was subjected to 10-fold serial dilutions (i.e., “equal gradient” refers to 10^−1^ to 10^−6^ dilutions using sterile PBS) to prepare concentration gradients, with absorbance measured at 600 nm to select a dilution with OD_600_ = 0.1 (corresponding to ~1 × 10^6^ CFU/mL, confirmed by plating 100 μL onto LB agar and counting colonies). For the inhibition assay, 100 μL of hydrogel extract (filtered through a 0.22 μm membrane), 50 μL of the calibrated bacterial solution, and 50 μL of fresh LB medium were added to each well of a 96-well plate and incubated at 37 °C for 4 h, 12 h, 24 h, and 48 h. Finally, the OD_600_ was measured to calculate the inhibition rate [19].

#### 2.6.2. Anti-Biofilm Test

The bacterial solution was adjusted to an optical density (OD) value of 1.0 using PBS. Then, 100 μL of extract, 50 μL of bacterial solution, and 50 μL of medium were added to each well of a 96-well aseptic cell culture plate aseptically on an ultra-clean bench. Six replicates were set up for each sample with 30-min incubation under standard laboratory conditions. The upper layer of solution was aspirated, and then the culture wells were washed 4 to 5 times with sterilized PBS to remove floating bacteria. Subsequently, 200 μL of methanol was added to each well for 30 min of fixation and then aspirated. Each well received 200 μL of 1% crystal violet solution and was incubated for 30 min at room temperature, before being rinsed five to six times with distilled water to remove excess stain. After drying, 200 μL of 33% acetic acid was added to each well, which was then incubated for 30 min to lyse the stained bacteria. Finally, absorbance was measured at 595 nm using an enzyme meter [25].

### 2.7. In Vitro Cellular Activity

#### 2.7.1. Biocompatibility

Human umbilical vein endothelial cells (HUVEC cells) were inoculated in the sterilized hydrogel extracts of each group in 96-well plates at an inoculum density of 2 × 10^4^ cells/well, and the 96-well plates were placed in an incubator under culture conditions of 37 °C and 5% CO_2_ for 24 h. The original medium was exchanged with 200 μL of serum-free DMEM cell culture medium supplemented with 10% CCK-8 reagent. Following 120-min incubation in the incubator, the liquid was transferred to a 96-well plate for absorbance measurement at 450 nm using an enzyme marker [25].

#### 2.7.2. Cell Migration

HUVEC cells were selected for cell migration assays to assess cell growth and migration after hydrogel treatment. Briefly, 3 × 10^4^ HaCaT cells per well were cultured in three 12-well plates separately until more than 90% confluence was reached. After scratching the cell surface with the tip of a 200 μL pipette gun, the cells were placed in an incubator at 37 °C with 5% CO_2_ and cultured using different groups of hydrogel infusions, and cell migration was observed by taking pictures with a microscope on days 1, 2, and 3 [26].

#### 2.7.3. Cell Viability

HUVECs were seeded in 96-well plates at a density of 1 × 10^4^ cells/well and maintained in hydrogel extract-supplemented medium at 37 °C with 5% CO_2_. The 96-well plates were kept at a temperature of 37 °C in a humidified incubator supplied with 5% CO_2_. An AO/EB mixed fluorescence staining solution was prepared by mixing equal amounts of acridine orange (AO) solution and ethidium bromide (EB) solution in equal concentrations. Following a 24-h cultivation period of HUVEC cells, an appropriate amount of trypsin was added to the 96-well plate to dissociate them. Then, 20 μL of HUVEC cell suspension was stained with the appropriate amount of AO/EB, and cellular morphology was examined using a fluorescence inverted microscope [26].

### 2.8. Animal Models of Infected Wounds

KM mice (male, 20 ± 0.2 g, 4 weeks old) were purchased from Jinan Pengyue Experimental Animal Breeding Co. (Jinan, China). Thirty-six mice were randomly divided into four groups, with nine mice in each group: negative control group (treated with gauze), positive control group, PTD group, and PTDP group. Approval for the animal experiments was obtained approval from the Ethics Committee for Animal Experiments of Shandong University (SYXK (Lu) 2020–0022). Before the start of the experiment, the mice were anaesthetized with chloral hydrate (3.5%, 10 μL/1 g body weight) via the intraperitoneal route. The hair on the back was shaved and the skin was disinfected before creating an open excision wound. Two circular full-thickness skin wounds (8 mm in diameter) were made on the dehaired skin of the mouse’s back, and 20 μL of *S. aureus* suspension (1 × 10^8^ CFU/mL) was inoculated into each wound site to establish a skin wound model of *S. aureus* infection. PTD and PTDP gels were placed over the wound site before disinfection. The positive group was SKIN KANG RUI brand wound dressing (model: 203205, Weihai Jierui Medical Products Co., Ltd., Weihai, China), which was subsequently fixed with a 3 M sterile patch (model: 1624W, 3M Company, St. Paul, MN, USA). The dressing was changed every 2 days [27]. The ImageJ software (version 1.8.0, National Institutes of Health (NIH), Bethesda, MD, USA) was used to estimate the wound healing rate as follows:

(3) $$Wound\;healing\;\left(\%\right)=\frac{D_{0}-D_{n}}{D_{0}}\times 100$$

where $D_{0}$ and $D_{n}$ represent the diameters of the initial and final wound areas, respectively.

### 2.9. Histological Analysis

Mice were euthanized on days 3, 7, and 14, with three mice in each group, and skin wound tissues were collected. The collected tissues were treated and subsequently fixed in a 4% paraformaldehyde solution. They were then dehydrated using an alcoholic solution, embedded in paraffin, and sectioned with a microtome. H&E, Masson, and immunohistochemistry staining were performed to observe the infiltrating inflammatory cells, the level of collagen deposition, and the regulation of cells in the infected skin tissues. All images were observed using a light microscope and quantified using ImageJ software [27,28,29].

### 2.10. Statistical Analysis

Data are presented as mean ± SD (*n* ≥ 3 independent replicates). Statistical significance was determined by one-way ANOVA followed by Tukey’s post hoc test. The significance of differences was treated as follows: * *p* < 0.05, ** *p* < 0.01, *** *p* < 0.001, **** *p* < 0.0001.

## 3. Results and Discussion

### 3.1. Preparation and Characterization of Hydrogels

In this study, a drug-loaded hydrogel with excellent mechanical properties, strong adhesiveness, and multifunctionality was prepared based on co-assembly. Polyvinyl alcohol (PVA) experiences molecular crystallization, porosity changes, and chain rearrangements during freeze-thawing, affecting its mechanical properties. Thus, the low-temperature freeze-thaw method was used to give PTDPs basic PVA hydrogel properties, and tea polyphenols (TP) were introduced via in situ synthesis. TP’s hydrogen bonds rapidly formed a stable cross-linked network with PVA [30]. After preliminary screening, four representative PVA/TP ratios (1:0.5, 1:0.75, 1:1, 1:2) were selected for further study.

**Figure 1:**
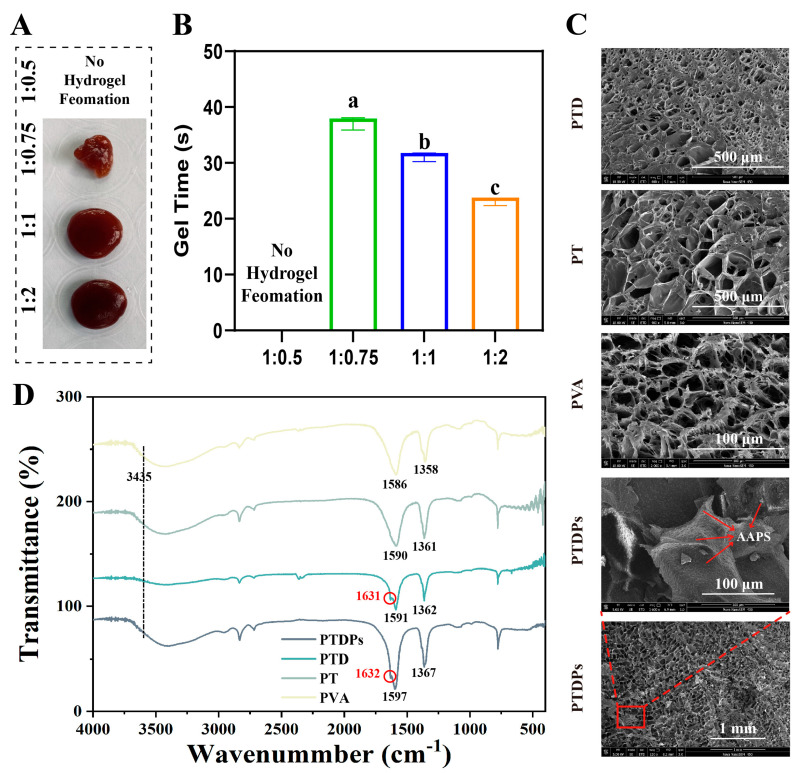
Preparation of hydrogels. (**A**) Gel formation for different PVA to TP ratios. (**B**) Gel formation time for different PVA to TP ratios (*n* = 3). (**C**) Cross-sectional scanning electron micrographs of PTD, PT, PVA, and PTDPs. (**D**) Infrared absorption spectra of different hydrogels. Note: The same superscript letters indicate no significant difference (*p* > 0.05), and different superscript letters indicate significant differences (*p* < 0.05).

The formation process of the co-assembled hydrogels was first studied by adjusting the mass ratios of each component. As shown in Figure 1A, when the mass ratio of PVA to TP was 1:0.5, no obvious hydrogel formation was observed in the system. This was due to the low content of TP, resulting in insufficient cross-linking density, which was unable to effectively connect PVA molecular chains to form a three-dimensional network structure. In contrast, stable and uniform hydrogels were successfully prepared at ratios of 1:0.75, 1:1, and 1:2, indicating that appropriately increasing the concentration of the cross-linking agent TP could significantly promote the gelation process and enhance the intermolecular cross-linking.

The gelation time of hydrogels with different component ratios was quantitatively measured (Figure 1B). During the experiment, the mixed solution was placed in a constant temperature environment (25 °C), and the gel formation state was monitored in real-time. The results showed that as the content of TP increased, the gelation time gradually decreased, from approximately 40 s at a ratio of 1:0.75 to 28 s at a ratio of 1:2. This indicated that a higher content of the cross-linking agent accelerated the network formation rate by enhancing intermolecular interactions, and statistical analysis confirmed significant differences among groups, further confirming the high sensitivity of gelation kinetics to formulation parameters (*p* < 0.05).

The microstructures of the hydrogels were observed via scanning electron microscopy (SEM) (Figure 1C). In PVA, PT, and PTD hydrogels, a relatively loose and disordered porous morphology was observed, with uneven pore sizes. In contrast, the PTDP hydrogel exhibited a more compact and organized porous network. Notably, within the pores of PTDPs, a large number of highly regular, nanoscale particulates could be clearly distinguished. These particulates corresponded to the incorporated marine self-assembling peptides (AAPs), indicating successful immobilization and uniform dispersion of AAPs within the hydrogel network. The presence of these ordered structures significantly increased the density of the porous framework, suggesting that AAPs not only participated in co-assembly but also regulated the microarchitecture at the nanoscale. Such highly organized microstructures are expected to enhance the mechanical integrity of the hydrogel and provide favorable cues for cell adhesion, migration, and proliferation during wound healing.

To further elucidate the molecular interactions during co-assembly, Fourier-transform infrared (FTIR) spectroscopy was performed (Figure 1D). The spectrum of PTDPs exhibited characteristic absorption peaks corresponding to O-H/N-H stretching (around 3435 cm^−1^), and amide I and II bands (around 1631 and 1586 cm^−1^, respectively). Compared to the spectra of PVA, PT, and PTD, the PTDP hydrogel showed distinct shifts in the amide I region (from 1632 cm^−1^ to 1631 cm^−1^) and enhanced peak intensities at 1358–1367 cm^−1^, suggesting the formation of strong hydrogen bonding and π-π stacking interactions among polyphenols, peptides, and PVA molecules. These spectral changes confirmed that polydopamine (PDA) introduced catechol groups forming dynamic covalent bonds (e.g., boronate esters) and hydrogen bonds with TP/PVA [31], while marine self-assembling peptides (AAPs) contributed amide groups to strengthen interchain interactions via hydrogen bonding and electrostatic forces.

In conclusion, the optimized PTDP hydrogel features rapid gelation, a stable network, and unique nano-microstructure via co-assembly of marine peptides and polyphenol networks. The gelation mechanism is rooted in the synergistic interplay of freeze-thaw-induced PVA crystallization, TP-mediated hydrogen bonding/π-π stacking, PDA-enhanced adhesive crosslinking, and AAPs-driven nanoscale scaffolding [18]. This multi-level crosslinking ensures mechanical robustness and endows dynamic adaptability for wound healing applications.

### 3.2. Plasticity and Adhesion

**Figure 2:**
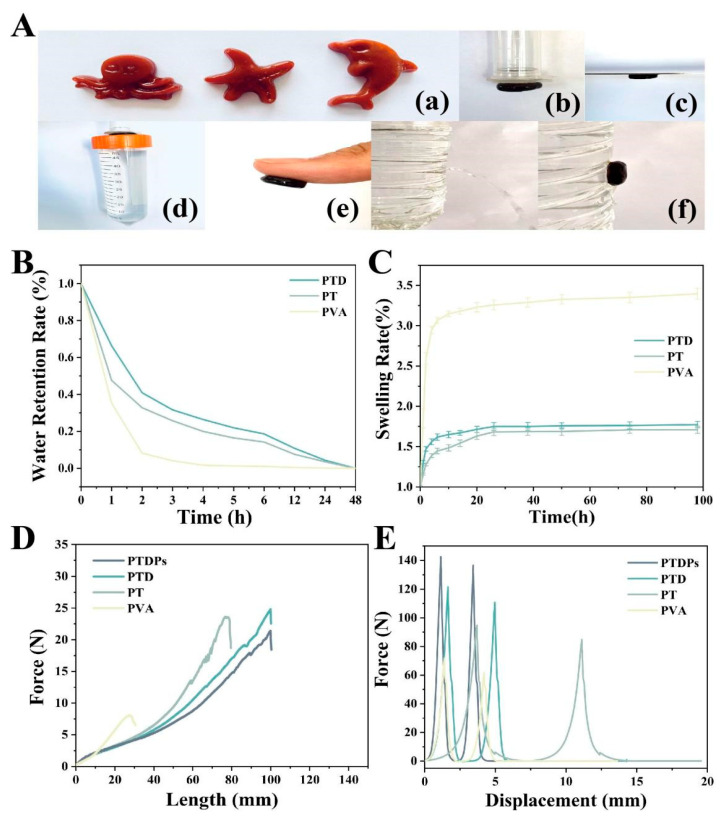
Properties of hydrogel. (**A**) Plasticity and adhesion (*n* = 3): (**a**) Actual picture of the plasticity of hydrogel; (**b**) Adhesion test of hydrogel to plastic material; (**c**) Adhesion of hydrogel to steel material; (**d**) Adhesion of hydrogel to heavy objects (60 g); (**e**) Adhesion of hydrogel to finger skin; (**f**) Diagram of hydrogel blocking test under wet environment. (**B**) Swelling behavior of hydrogel; (**C**) Water retention of hydrogel; (**D**) Tensile stress-displacement curve of hydrogel; (**E**) Compressive stress-displacement curve of hydrogel.

Hydrogels inherently exhibit high deformability, enabling them to undergo mechanical deformation (e.g., stretching, compression, extrusion) without compromising their structural integrity. This plasticity makes them suitable for biomedical applications where mimicking the softness and compliance of native tissues is essential for effective wound coverage and healing [32,33]. The PTDP hydrogel exhibited remarkable moldability, as shown in Figure 2A(a), where the hydrogel precursors were shaped into complex geometries and retained their form post-gelation. Tensile testing (Figure 2D) revealed that PTDPs could stretch to 2.85 times their original length, with a maximum tensile force of 23.8 N. This elongation capacity exceeded that of PVA (1.5-fold, 7.8 N), PT (2.1-fold, 18.5 N), and PTD (2.7-fold, 24.8 N) hydrogels, indicating enhanced deformability.

The adhesive behavior of hydrogels is primarily governed by the presence of reactive functional groups such as hydroxyl (-OH) and amine (-NH_2_) moieties, which mediate hydrogen bonding, electrostatic interactions, and van der Waals forces with target surfaces [34]. Notably, polydopamine (PDA), inspired by mussel foot proteins, introduces catechol groups that form dynamic covalent and non-covalent interactions with organic and inorganic substrates. The adhesive properties of PTDPs were evaluated against various substrates. As depicted in Figure 2A(b–f), the hydrogel adhered firmly to plastic, steel, and porcine skin, sustaining a 63 g load without detachment (Figure 2A(d)). Under wet conditions (90% humidity), PTDPs effectively sealed against water flow (Figure 2A(f)), outperforming PVA and PT hydrogels. Qualitative analysis via image-based assessment showed that PTDPs exhibited the strongest adhesion among all groups, consistent with the presence of polydopamine (PDA) which mediates catechol-based interactions.

The improved plasticity and adhesion of the PTDP hydrogel could be attributed to the synergistic non-covalent interactions among PVA, TP, PDA, and AAPs. Hydrogen bonding between hydroxyl-rich TP and PVA, combined with π-π stacking involving TP and AAPs, facilitated rapid co-assembly into a densely crosslinked network. Moreover, the freeze-thaw cycling induced PVA crystallite formation, enhancing matrix elasticity and mechanical resilience.

### 3.3. Swelling Rate and Water Retention Rate

In the hydrogels prepared in this study, the PVA, TP, and PDA used all had strong hydrophilicity, but since the number and proportion of hydrophilic groups dominated by hydroxyl groups in the molecular chain of PVA were much higher than those of TP and PDA, the control PVA hydrogel exhibited stronger interactions with water molecules, which, in turn, was reflected in a stronger solubilization behavior (Figure 2B). PTDPs reached an equilibrium swelling ratio of 220% within 24 h, lower than PVA (310%) but higher than the PT (250%) and PTD (230%) hydrogels. The reduced swelling in PTDPs was attributed to the denser crosslinking network formed by tea polyphenols (TP) and PDA, as observed in SEM images (Figure 1C).

PTDPs demonstrated superior water retention, maintaining 25% moisture content after 6 h (Figure 2C), i.e., significantly higher than PVA (15%), PT (20%), and PTD (22%) hydrogels. This property was attributed to the hydrophilic nature of PVA and the hydrogen-bonding capacity of TP and self-assembling peptides (AAPs), which stabilized water molecules within the network.

### 3.4. Mechanical Properties of Hydrogels

Hydrogel dressings experience dynamic mechanical stresses during wound healing, including tissue shear forces and environmental fluctuations. Therefore, a comprehensive evaluation of the morphological changes of hydrogels under tensile and compressive stresses is essential [35]. In our study, tensile stress-displacement curves (Figure 2D) showed that PTDPs had a tensile modulus of 0.8 MPa and a breaking strength of 1.2 MPa, surpassing PVA (0.5 MPa, 0.3 MPa) and PT (0.7 MPa, 0.9 MPa) hydrogels. The maximum tensile force of 23.8 N for PTDPs was slightly lower than PTD (24.8 N) but significantly higher than PVA and PT, likely due to the inclusion of AAPs that slightly disrupted the PVA-TP network.

Compressive testing (Figure 2E) revealed that PTDPs withstood a maximum force of 142.5 N at 50% strain, exceeding PT (110.3 N) and PVA (55.6 N) hydrogels. The stress–strain curve indicated that PTDPs had a compressive modulus of 1.5 MPa, closely matching the mechanical properties of human skin (0.5–1.5 MPa), making them suitable for dynamic wound environments.

Overall, relative to conventional freeze-thaw crosslinked PVA hydrogels, the hydrogels formed mainly by PVA and TP through hydrogen bonding interactions had stronger tensile properties, hardness, elasticity, etc. This proves that the degree of denseness of the crosslinked network of the hydrogels reflects the strength of the mechanical characteristics to a certain extent. This implies that PVA/TP-type hydrogels could be more adaptable to physiological activities such as human movement, skin stretching, and tissue repair in practical applications.

### 3.5. In Vitro Antioxidant Activity of Hydrogels

**Figure 3:**
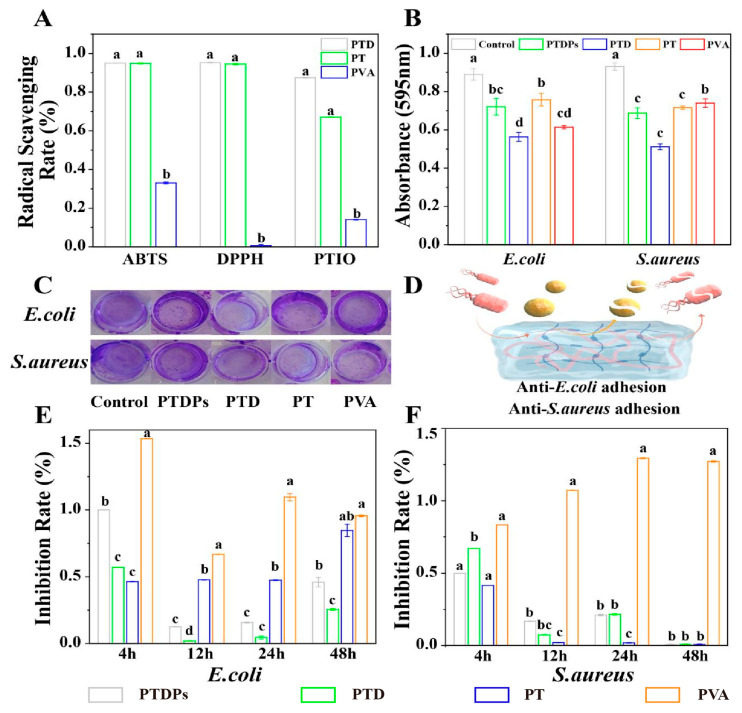
In vitro activities of hydrogels (*n* = 3). (**A**) Antioxidant activity evaluated by ABTS, DPPH, and PTIO radical scavenging assays; (**B**) Quantitative absorbance statistics of crystal violet staining for assessing bacterial biofilm formation; (**C**) Representative real-time images of crystal violet staining; (**D**) Schematic diagram of antibacterial effect of hydrogel; (**E**) Inhibition rate of hydrogels against *E. coli*; (**F**) Inhibition rate of hydrogels against *S. aureus*. Note: The same superscript letters indicate no significant difference (*p* > 0.05), and different superscript letters indicate significant differences (*p* < 0.05).

The antioxidant capacity of hydrogels was evaluated using ABTS, DPPH, and PTIO radical scavenging assays (Figure 3A). PTD and PT hydrogels exhibited robust scavenging efficiency against ABTS and DPPH radicals, with clearance rates exceeding 93% for both assays. PTD hydrogel demonstrated a significantly higher PTIO radical scavenging rate of 86% compared to PT (65%) and PVA (12%) (*p* < 0.01). The PTDP hydrogel, incorporating PDA and AAPs, showed scavenging rates of 94% (ABTS), 93.5% (DPPH), and 82% (PTIO), indicating that PDA enhances the hydrogel’s responsiveness to diverse free radicals.

The superior antioxidant activity of PTD and PTDPs was attributed to the polyphenolic structure of TP and the catechol groups of PDA, which donated hydrogen atoms to quench free radicals [30]. The PTIO assay, specific for hydroxyl radicals, revealed that PDA improved the hydrogel’s ability to scavenge reactive oxygen species (ROS) in hypoxic wound environments. The immediate antioxidant effect (measured within 24 h of hydrogel formation) suggested that PTDPs can mitigate oxidative stress during the initial inflammatory phase, preventing ROS-mediated degradation of growth factors and extracellular matrix components. This property is critical for infected wounds, where excessive ROS impairs epithelial migration and angiogenesis [36]. Thus, reducing the risk of infection associated with excess ROS during the inflammatory phase [37].

### 3.6. In Vitro Antibacterial Activity of Hydrogels

Antibacterial assays showed that PTDPs significantly inhibited *E. coli* and *S. aureus* growth, with inhibition rates below 25% at 12 h and complete suppression of *S. aureus* at 48 h (Figure 3E,F). In contrast, the PVA hydrogel promoted bacterial proliferation, with OD600 values 1.3-fold higher than the blank control, highlighting the necessity of PVA modification. Crystal violet staining (Figure 3B,C) revealed that PTD exhibited the strongest biofilm inhibition, reducing *E. coli* and *S. aureus* biomass by 70% and 65%, respectively, compared to the control (*p* < 0.05). PTDPs showed comparable biofilm inhibition to PTD (68% for *E. coli*, 63% for *S. aureus*), indicating that AAPs did not compromise the antibacterial effect of PDA-TP networks. The antibacterial mechanism schematic (Figure 3D) illustrates that TP disrupted bacterial membranes via hydrophobic interactions, while PDA generated ROS to induce oxidative damage, demonstrating the synergistic action of dual components.

The antibacterial efficacy of PTDPs stemmed from the synergistic action of TP and PDA, as visualized in Figure 3D. TP disrupts bacterial membranes via hydrophobic interactions, while PDA’s catechol groups generate ROS to induce oxidative damage [11]. The complete inhibition of *S. aureus* at 48 h (Figure 3F) suggested that PTDPs can combat both planktonic bacteria and biofilm-forming pathogens, which are major causes of chronic wound infections [38]. The observation that PVA promoted bacterial growth underscores the importance of incorporating antibacterial components (TP/PDA) to prevent wound reinfection. These results align with the hydrogel’s design: PDA provides broad-spectrum antimicrobial activity, while AAPs maintain pro-regenerative functions without compromising efficacy. The consistent biofilm inhibition between PTD and PTDPs (Figure 3B,C) confirmed that AAP integration did not interfere with the antibacterial network, supporting the feasibility of multi-component co-assembly.

### 3.7. In Vitro Cell Viability of Hydrogels

**Figure 4:**
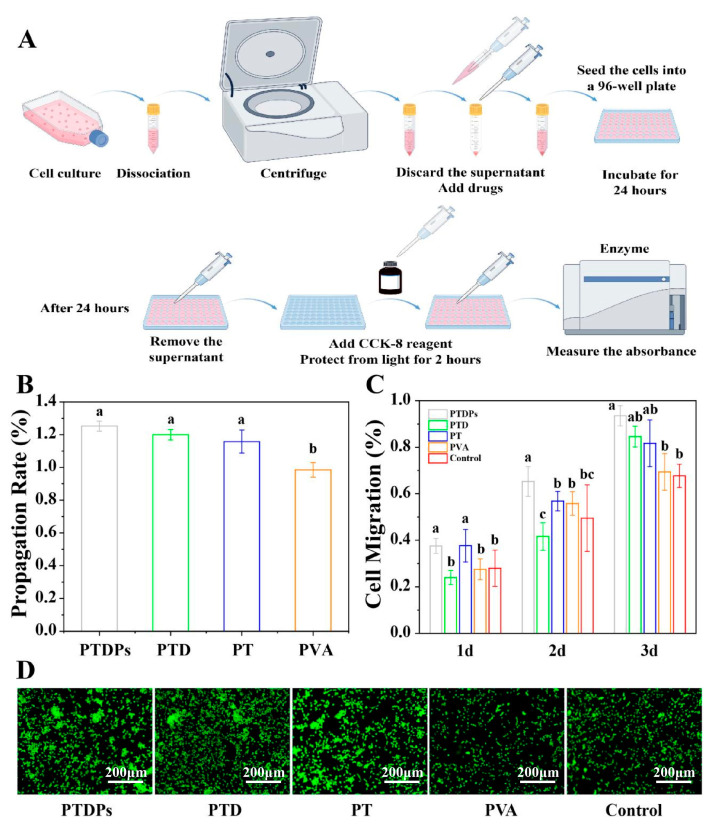
Cytotoxicity and cell proliferation of hydrogels (*n* = 3). (**A**) Schematic diagram of the CCK-8 assay procedure for evaluating cell viability; (**B**) Quantitative results of CCK-8 assay showing cell viability after 24 h co-culture with hydrogels; (**C**) Cell migration rates of HUVECs treated with different hydrogels at 1, 2, and 3 days, as measured by scratch wound healing assay; (**D**) AO/EB staining images of HUVECs after 24 h treatment, showing live cells (green fluorescence) and dead cells (red fluorescence). Note: The same superscript letters indicate no significant difference (*p* > 0.05), and different superscript letters indicate significant differences (*p* < 0.05).

Excellent biocompatibility is a basic requirement for hydrogel wound dressings. In this study, the toxicity of the hydrogels was tested by CCK-8 activity assay (Figure 4A,B). The results showed that the cell viability was higher than 100% in all experimental groups except for the PVA hydrogel, with more than 120% in the PTDP group. This proved that the combination of PVA, PT, and PDA was a green and safe non-covalent cross-linking, which did not produce substances with cytotoxicity, while the hydrogels were compatible with the cell proliferation-promoting properties of AAPs, which gave them the ability to promote tissue repair.

Cell migration represents a fundamental cellular behavior and serves as a physiological process essential for the normal growth and development of the body. It constitutes a vital form of movement commonly observed in living cells. A high cell migration rate implies a greater ability to form tissues during the wound healing process and enable faster repair. As shown in Figure 4C, the migration rate of the PTDP hydrogel with AAPs peptides was markedly higher than that of the other groups in the first, second, and third days, reaching as high as 93.5% on the third day, which was nearly 26% higher than that of the blank control group. More interestingly, PTDs, although they did not show strong cell migration on days 0–2, jumped to the second-highest cell migration rate on the third day.

The results of AO/EB staining (Figure 4D) show that the cells in all groups were stained fluorescent green, which proved that the cells were in good condition, and corroborated the CCK-8 results, which proved that the series of hydrogels were safe for the cells and had specific growth-promoting effects.

### 3.8. Hydrogel Treatment of S. aureus Infected Wounds in the Whole Cortex of Mice

**Figure 5:**
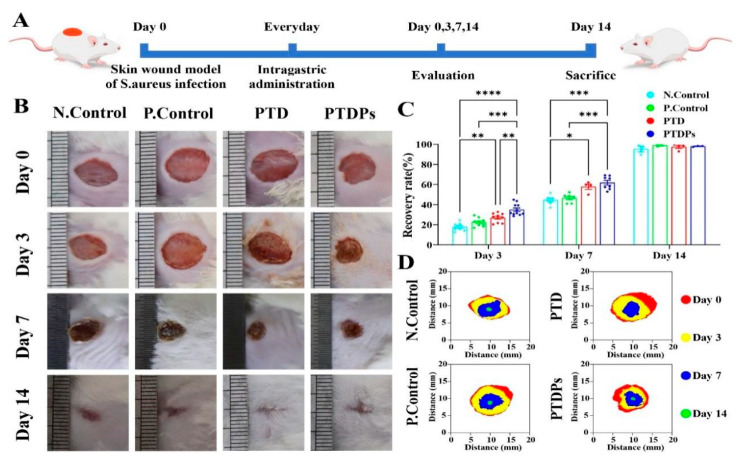
PTDP hydrogel treatment of *S. aureus* infected wounds in the whole cortex of mice (*n* = 3). (**A**) Schematic diagram of the experimental procedure for treating whole cortex *S. aureus*-infected wounds in mice; (**B**) Images of infected wounds in each group of treated mice from day 0, 3, 7, and 14; (**C**) Quantitative results of the wound healing; (**D**) Superimposed graphs of the area of the wound area at different time points, with the colors from the outside in order of days 0, 3, 7 and 14. Note: Asterisks denote statistical significance compared to the control group: * *p* < 0.05, ** *p* < 0.01, *** *p* < 0.001, **** *p* < 0.0001.

To investigate the role of the PTDP hydrogel in promoting wound healing in vivo, a mouse whole cortex wound model was constructed (Figure 5A). Wound healing areas were photographed and quantitatively analyzed on days 0, 3, 7, and 14 after injury (Figure 5B–D). On postoperative days 3 and 7, the PTDP group demonstrated significant wound healing, with wound healing rates of 34.99 ± 5.54% and 61.86 ± 6.11%, respectively. The PTDPs demonstrated a statistically significant difference when compared to the Control group (** *p* < 0.01). When the wounds had healed well in all groups on postoperative day 14, their healing rates were close to 100%. The Control group, however, still had a few wounds (95.38 ± 4.12%). The experimental results showed that PTDP hydrogel significantly accelerated healing kinetics in bacterially infected wounds.

### 3.9. Histologic Assessment of Wound Tissue

**Figure 6:**
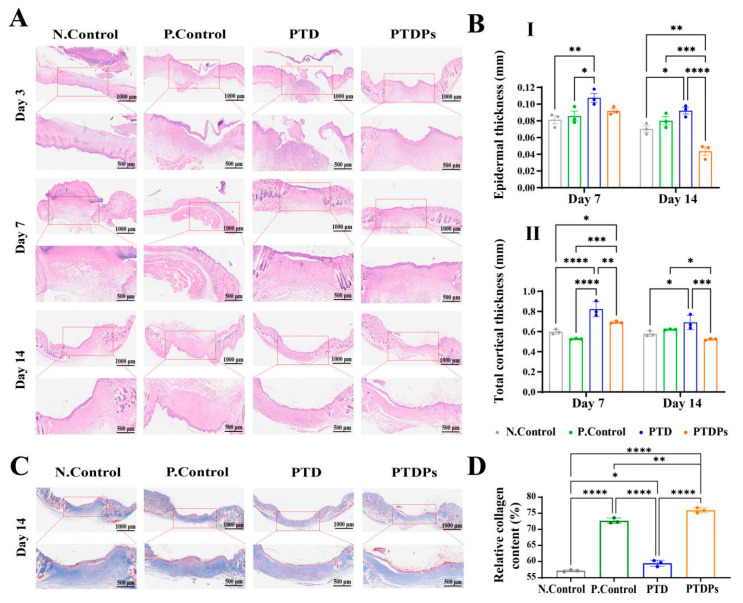
Histologic assessment of wound tissue (*n* = 3). (**A**) Representative images of H&E staining; (**B**) I. Epidermal thickness; II. Total cortical thickness. (**C**) Representative images of Masson staining; (**D**) Collagen content of skin wounds on day 14. Note: Asterisks denote statistical significance compared to the control group: * *p* < 0.05, ** *p* < 0.01, *** *p* < 0.001, **** *p* < 0.0001.

Wound healing comprises a complex cascade of physiological processes, including inflammatory response, cell migration, and tissue remodeling [39]. H&E staining, as shown in Figure 6A, was employed to characterize the details of wound healing processes. On day 3, wounds in all groups showed substantial inflammation and fibroblast aggregation. After a period of 7 days, wounds treated with hydrogels exhibited enhanced re-epithelialization in comparison to the control group. Notably, wounds treated with the PTDP hydrogel exhibited significantly diminished inflammatory cell infiltration, suggesting accelerated resolution of the acute inflammatory phase. This anti-inflammatory effect likely stemmed from the antimicrobial and antioxidant properties of PTD hydrogels, which were further enhanced by AAPs. These properties may inhibit bacterial colonization during the early stages of wound healing, thereby reducing inflammation. After 14 days of treatment, epithelial chemotaxis was complete in all groups of hydrogels, but the PTDP hydrogel-treated wounds showed a more regular epithelial layer. Notably, the epidermal thickness and total thickness of the cortex were significantly lower in PTDPs. This suggests that the PTDP hydrogel achieved better performance in terms of accelerating infected wound healing compared to other groups.

In addition, collagen deposition and maturation during wound healing were evaluated via Masson [40]. The results showed (Figure 6C) that collagen deposition was significantly higher in the P. Control (** *p* < 0.01) and PTDP (** *p* < 0.01) groups than in the N. Control group. Specifically, the collagen content in the N. Control, P. Control, PTD, and PTDP groups was 57.15 ± 0.25%, 72.66 ± 0.67%, 59.44 ± 0.61% and 75.92 ± 0.66%, respectively (Figure 6D), and the PTDP group showed the highest level of collagen deposition, which was characterized by a collagen fiber that was more organized, with a regular arrangement to increase collagen deposition. This suggests that the PTDP hydrogel have antimicrobial ability which could demonstrate significant therapeutic efficacy in bacterially infected wound models, as well as concurrently mitigating local inflammation and further enhancing collagen secretion and skin regeneration, thus greatly improving the healing rate of infected wounds.

### 3.10. Immunohistochemistry

**Figure 7:**
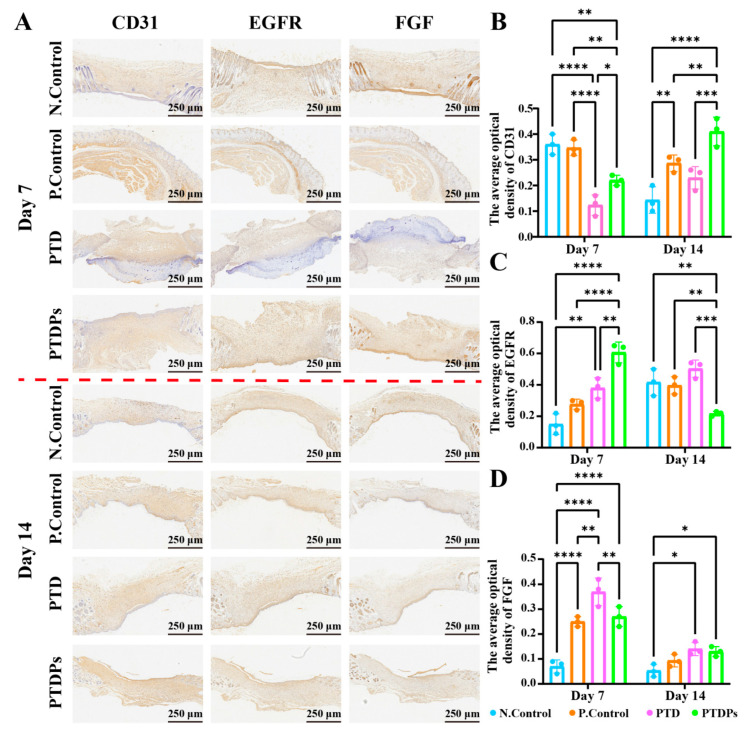
Immunohistochemistry of wound tissue after 7 and 14 days of different hydrogel treatments (*n* = 3). (**A**) Immunohistochemical images of wound tissues stained for CD31, EGFR, and FGF after 7 and 14 days of different hydrogel treatments; (**B**–**D**) Quantitative statistics of relative expression after 7 and 14 days of different hydrogel treatments. Note: Asterisks denote statistical significance compared to the control group: * *p* < 0.05, ** *p* < 0.01, *** *p* < 0.001, **** *p* < 0.0001.

Immunohistochemical analysis was conducted to evaluate key markers involved in angiogenesis, epithelial proliferation, and tissue remodeling (Figure 7A). CD31, a classical marker for endothelial cells and neovascularization, is widely used to assess micro-vessel density and vascular remodeling during wound repair [41]. As shown in Figure 7B, on day 7, the PTDP group exhibited significantly lower CD31 expression compared to both the N. Control (** *p* < 0.01) and P. Control (** *p* < 0.01) groups, but higher than the PTD group (* *p* < 0.05), suggesting delayed but progressive angiogenesis. By day 14, CD31 expression in the PTDP group was significantly elevated over all other groups (** *p* < 0.01), indicating enhanced neovascularization facilitated by the hydrogel, likely due to the nano-fibrous network self-assembled by AAPs, which mimics the extracellular matrix (ECM) to provide a physical scaffold for endothelial cell migration and vascular sprouting.

EGFR, a receptor tyrosine kinase involved in keratinocyte proliferation, differentiation, and re-epithelialization during wound healing [42], showed strong upregulation in the PTDP group at day 7 (Figure 7C), with expression levels significantly exceeding those in the N. Control, P. Control, and PTD groups (** *p* < 0.01). However, EGFR expression decreased markedly by day 14, suggesting that the hydrogel maintained local microenvironmental stability through the hydrogen bond network formed by TP and PVA, and promoted early-stage epithelial proliferation and differentiation by releasing antioxidant components to inhibit inflammatory factor-mediated damage to epithelial cells during the repair process.

FGF, a key mediator of fibroblast and keratinocyte proliferation and angiogenesis, followed a similar temporal trend (Figure 7D). At day 7, FGF expression in the PTDP group was intermediate-higher than the N. Control but lower than the PTD and P. Control groups. Notably, by day 14, PTDP-treated wounds exhibited the highest FGF levels, indicating a sustained stimulatory effect on tissue remodeling and matrix regeneration.

Collectively, these findings demonstrate that the PTDP hydrogel promotes coordinated regulation of angiogenesis, epidermal regeneration, and fibroblast activation, thereby accelerating and enhancing wound healing in infected conditions.

## 4. Conclusions

This study successfully demonstrated the rational design and fabrication of a multifunctional hydrogel based on a bioinspired co-assembly strategy. The integration of PVA, TP, PDA, and AAPs resulted in a biomaterial with superior mechanical strength, dynamic adhesion, and multifaceted biological functions. In vitro evaluations confirmed the PTDP hydrogel’s remarkable antioxidant, antimicrobial, and pro-regenerative properties, while in vivo experiments in an infected wound model validated its efficacy in terms of modulating the wound microenvironment and promoting tissue regeneration. These results not only establish the PTDP hydrogel as a viable solution for managing infected wounds but also highlight the substantial potential of supramolecular assembly strategies in the development of advanced regenerative biomaterials. By bridging the gap between antimicrobial functionality and tissue regenerative capacity, this study provides valuable insights and a novel paradigm for the design of next-generation bioactive hydrogels, paving the way for further advancements in biomaterial science and wound healing applications. Future research could focus on optimizing the hydrogel formulation for large-scale production and conducting pre-clinical studies to fully explore its translational potential.

## Data Availability

Data supporting the reported results are contained within the article or are available from the corresponding author.

## References

[1] Han G., Ceilley R. (2017). Chronic Wound Healing: A Review of Current Management and Treatments. Adv. Ther..

[2] Wu Y.-K., Cheng N.-C., Cheng C.-M. (2019). Biofilms in Chronic Wounds: Pathogenesis and Diagnosis. Trends Biotechnol..

[3] Edwards R., Harding K.G. (2004). Bacteria and Wound Healing. Curr. Opin. Infect. Dis..

[4] Liang Y., He J., Guo B. (2021). Functional Hydrogels as Wound Dressing to Enhance Wound Healing. ACS Nano.

[5] Xu N., Ma N., Yang X., Ling G., Yu J., Zhang P. (2020). Preparation of Intelligent DNA Hydrogel and Its Applications in Biosensing. Eur. Polym. J..

[6] Huang M., Cai D., Liu Y., Sun J., Wang J., Qin C., Dai L., Kazuo Y. (2012). Investigation of A-PVA/s-PVA Hydrogels Prepared by Freezing-Thawing Method. Fibers Polym..

[7] Zhou Z., Chen Z., Ji C., Wu C., Li J., Ma Y., Jin S., Fang X., Wu Y., Xun J. (2024). A Dopamine-Assisted Antioxidative in Situ-Forming Hydrogel with Photothermal Therapy for Enhancing Scarless Burn Wound Healing. Chem. Eng. J..

[8] Cai J., Liu S., Zhong Q., Shang Y., Chen Z., Yao Y., Zhou B., Yin F., Zhao J., Zheng L. (2024). Multifunctional PDA/ZIF8 Based Hydrogel Dressing Modulates the Microenvironment to Accelerate Chronic Wound Healing by ROS Scavenging and Macrophage Polarization. Chem. Eng. J..

[9] Zhong Y., Lin Q., Yu H., Shao L., Cui X., Pang Q., Zhu Y., Hou R. (2024). Construction Methods and Biomedical Applications of PVA-Based Hydrogels. Front. Chem..

[10] Calo E., Barros J., Ballamy L., Khutoryanskiy V.V. (2016). Poly(Vinyl Alcohol)-Gantrez^®^ AN Cryogels for Wound Care Applications. RSC Adv..

[11] Chinchulkar S.A., Patra P., Dehariya D., Yu A., Rengan A.K. (2022). Polydopamine Nanocomposites and Their Biomedical Applications: A Review. Polym. Adv. Technol..

[12] Liu Z., Qu S., Weng J. (2015). Application of Polydopamine in Surface Modification of Biomaterials. Prog. Chem..

[13] Whitesides G.M., Grzybowski B. (2002). Self-Assembly at All Scales. Science.

[14] Liu Y., Ma Q., Tang L., Shen Y., Zhao H., Liu X., Lin D., Zhou G. (2024). A Multifunctional Hydrogel with Mild Photothermal Antibacterial and Antioxidant Properties Based on Quercetin and Dopamine-Coated Zinc Oxide Nanoparticles for Healing Bacteria-Infected Wound. Chem. Eng. J..

[15] Yang F., Zhao D., Zhang K., Wang Z., Wang Y., Wu C., Cui S., Guo T., Chen L., Chen J. (2022). Oral Delivery of Marine Shellfish Supramolecule Peptides for Skin Wound Healing. Colloids Surf. B-Biointerfaces.

[16] Zeng H., Tang L., Huang L., Yang N., Chen X., Peng X., Chen Z., Guo J., Weng J., Guo T. (2025). A Novel Multi-Functional PVA- Alginate Hydrogel with Dynamic Bond Crosslinking for Infected Wound Repair. Carbohydr. Polym..

[17] Kamoun E.A., Kenawy E.-R.S., Tamer T.M., El-Meligy M.A., Eldin M.S.M. (2015). Poly (Vinyl Alcohol)-Alginate Physically Crosslinked Hydrogel Membranes for Wound Dressing Applications: Characterization and Bio-Evaluation. Arab. J. Chem..

[18] Huang Q., Hu Y., Chen Y., Zhou M., Zhang Y., Sun Z., Chen Z. (2025). An Antimicrobial and Adhesive Conductive Chitosan Quaternary Ammonium Salt Hydrogel Dressing for Combined Electrical Stimulation and Photothermal Treatment to Promote Wound Healing. Carbohydr. Polym..

[19] Liu X., Sun Y., Wang J., Kang Y., Wang Z., Cao W., Ye J., Gao C. (2024). A Tough, Antibacterial and Antioxidant Hydrogel Dressing Accelerates Wound Healing and Suppresses Hypertrophic Scar Formation in Infected Wounds. Bioact. Mater..

[20] Zhao X., Liang Y., Huang Y., He J., Han Y., Guo B. (2020). Physical Double-Network Hydrogel Adhesives with Rapid Shape Adaptability, Fast Self-Healing, Antioxidant and NIR/pH Stimulus-Responsiveness for Multidrug-Resistant Bacterial Infection and Removable Wound Dressing. Adv. Funct. Mater..

[21] Tu C., Lu H., Zhou T., Zhang W., Deng L., Cao W., Yang Z., Wang Z., Wu X., Ding J. (2022). Promoting the Healing of Infected Diabetic Wound by an Anti-Bacterial and Nano-Enzyme-Containing Hydrogel with Inflammation-Suppressing, ROS-Scavenging, Oxygen and Nitric Oxide-Generating Properties. Biomaterials.

[22] Chandika P., Kim M.-S., Khan F., Kim Y.-M., Heo S.-Y., Oh G.-W., Kim N.G., Jung W.-K. (2021). Wound Healing Properties of Triple Cross-Linked Poly (Vinyl Alcohol)/Methacrylate Kappa-Carrageenan/Chitooligosaccharide Hydrogel. Carbohydr. Polym..

[23] Tang F., Miao D., Huang R., Zheng B., Yu Y., Ma P., Peng B., Li Y., Wang H., Wu D. (2024). Double-Layer Asymmetric Porous Mesh with Dynamic Mechanical Support Properties Enables Efficient Single-Stage Repair of Contaminated Abdominal Wall Defect. Adv. Mater..

[24] Xiong S., Li R., Ye S., Ni P., Shan J., Yuan T., Liang J., Fan Y., Zhang X. (2022). Vanillin Enhances the Antibacterial and Antioxidant Properties of Polyvinyl Alcohol-Chitosan Hydrogel Dressings. Int. J. Biol. Macromol..

[25] Ouyang J., Bu Q., Tao N., Chen M., Liu H., Zhou J., Liu J., Deng B., Kong N., Zhang X. (2022). A Facile and General Method for Synthesis of Antibiotic-Free Protein-Based Hydrogel: Wound Dressing for the Eradication of Drug-Resistant Bacteria and Biofilms. Bioact. Mater..

[26] Sun M., Tian Y., Liu J., Yan Y., Zhang X., Xiao C., Jiang R. (2024). Proanthocyanidins-Based Tandem Dynamic Covalent Cross-Linking Hydrogel for Diabetic Wound Healing. Int. J. Biol. Macromol..

[27] Zou C.-Y., Lei X.-X., Hu J.-J., Jiang Y.-L., Li Q.-J., Song Y.-T., Zhang Q.-Y., Li-Ling J., Xie H.-Q. (2022). Multi-Crosslinking Hydrogels with Robust Bio-Adhesion and pro-Coagulant Activity for First-Aid Hemostasis and Infected Wound Healing. Bioact. Mater..

[28] Yang Y., Liang Y., Chen J., Duan X., Guo B. (2022). Mussel-Inspired Adhesive Antioxidant Antibacterial Hemostatic Composite Hydrogel Wound Dressing via Photo-Polymerization for Infected Skin Wound Healing. Bioact. Mater..

[29] Shiekh P.A., Singh A., Kumar A. (2020). Exosome Laden Oxygen Releasing Antioxidant and Antibacterial Cryogel Wound Dressing OxOBand Alleviate Diabetic and Infectious Wound Healing. Biomaterials.

[30] Ke T., Zhao L., Fan X., Gu H. (2023). Rapid Self-Healing, Self-Adhesive, Anti-Freezing, Moisturizing, Antibacterial and Multi-Stimuli-Responsive PVA/Starch/Tea Polyphenol-Based Composite Conductive Organohydrogel as Flexible Strain Sensor. J. Mater. Sci. Technol..

[31] Zheng D., Huang C., Hu Y., Zheng T., An J. (2022). Constructions of Synergistic Photothermal Therapy Antibacterial Hydrogel Based on Polydopamine, Tea Polyphenols and Polyvinyl Alcohol and Effects on Wound Healing in Mouse. Colloids Surf. B Biointerfaces.

[32] Le X., Lu W., Zhang J., Chen T. (2019). Recent Progress in Biomimetic Anisotropic Hydrogel Actuators. Adv. Sci..

[33] Yang J., Wang S. (2023). Polysaccharide-Based Multifunctional Hydrogel Bio-Adhesives for Wound Healing: A Review. Gels.

[34] Yang J., Bai R., Chen B., Suo Z. (2020). Hydrogel Adhesion: A Supramolecular Synergy of Chemistry, Topology, and Mechanics. Adv. Funct. Mater..

[35] Khodaei T., Nourmohammadi J., Ghaee A., Khodaii Z. (2023). An Antibacterial and Self-Healing Hydrogel from Aldehyde-Carrageenan for Wound Healing Applications. Carbohydr. Polym..

[36] Cui S., Yang F., Yu D., Shi C., Zhao D., Chen L., Chen J. (2023). Double Network Physical Crosslinked Hydrogel for Healing Skin Wounds: New Formulation Based on Polysaccharides and Zn^2+^. Int. J. Mol. Sci..

[37] Hirtzel J., Leks G., Favre J., Frisch B., Talon I., Ball V. (2023). Strongly Metal-Adhesive and Self-Healing Gelatin@Polydopamine-Based Hydrogels with Long-Term Antioxidant Activity. Antioxidants.

[38] Li M., Xiao H., Su Y., Cheng D., Jia Y., Li Y., Yin Q., Gao J., Tang Y., Bai Q. (2023). Synergistic Inhibitory Effect of Honey and Lactobacillus Plantarum on Pathogenic Bacteria and Their Promotion of Healing in Infected Wounds. Pathogens.

[39] Guo B., Dong R., Liang Y., Li M. (2021). Haemostatic Materials for Wound Healing Applications. Nat. Rev. Chem..

[40] Wang N., Liu W., Chai G., Sun S., Ding Q., Cheng Z., Liu X., Zhao Y., Zhao T., Wang Y. (2023). Antibacterial, Anti-Inflammatory, Rapid Hemostasis, and Accelerated Repair by Multifunctional Metal–Organic Frameworks Fibrous Scaffolds for Diabetic Wounds. Chem. Eng. J..

[41] Chai G., Wang N., Xu M., Ma L., Liu X., Ding Q., Zhang S., Li A., Xia G., Zhao Y. (2024). Poly (Vinyl Alcohol)/Sodium Alginate/Carboxymethyl Chitosan Multifunctional Hydrogel Loading HKUST-1 Nanoenzymes for Diabetic Wound Healing. Int. J. Biol. Macromol..

[42] Yu D., Cui S., Chen L., Zheng S., Zhao D., Yin X., Yang F., Chen J. (2023). Marine-Derived Bioactive Peptides Self-Assembled Multifunctional Materials: Antioxidant and Wound Healing. Antioxidants.

